# Production and Immunogenicity Assessment of LTp50: An *Escherichia coli*-Made Chimeric Antigen Targeting S1- and S2-Epitopes from the SARS-CoV-2/BA.5 Spike Protein

**DOI:** 10.3390/ph17030302

**Published:** 2024-02-27

**Authors:** Alejandra Wong-Arce, Omar Gonzalez-Ortega, Andrea Romero-Maldonado, Arleth Miranda-López, Mariano García-Soto, Susan Farfán-Castro, Lourdes Betancourt-Mendiola, Samaporn Teeravechyan, Kanjana Srisutthisamphan, Mauricio Comas-García, Karla I. Solís Andrade, Sergio Rosales-Mendoza

**Affiliations:** 1 Facultad de Ciencias Químicas, Universidad Autónoma de San Luis Potosí, Av. Dr. Manuel Nava 6, San Luis Potosí 78210, Mexico; alejandra.wong@uaslp.mx (A.W.-A.); andrea.romma@gmail.com (A.R.-M.); a317919@alumnos.uaslp.mx (A.M.-L.); mariano.soto@uaslp.mx (M.G.-S.); a205029@alumnos.uaslp.mx (S.F.-C.); lourdes.betancourt@uaslp.mx (L.B.-M.); a290733@alumnos.uaslp.mx (K.I.S.A.); 2 Sección de Biotecnología, Centro de Investigación en Ciencias de la Salud y Biomedicina, Universidad Autónoma de San Luis Potosí, Av. Sierra Leona 550, Lomas 2a Sección, San Luis Potosí 78210, Mexico; 3 Virology and Cell Technology Laboratory, National Center for Genetic Engineering and Biotechnology, National Science and Technology Development Agency, 113 Thailand Science Park Phaholyothin Rd, Klong 1, Klong Luang, Pathumthani 12120, Thailand; samaporn.tee@biotec.or.th (S.T.); kanjana.sri@biotec.or.th (K.S.); 4 Sección de Microscopía de Alta Resolución, Centro de Investigación en Ciencias de la Salud y Biomedicina, Universidad Autónoma de San Luis Potosí, Av. Sierra Leona 550, Lomas 2a Sección, San Luis Potosí 78210, Mexico; mauricio.comas@uaslp.mx; 5 Sección de Medicina Molecular y Translacional, Centro de Investigación en Ciencias de la Salud y Biomedicina, Universidad Autónoma de San Luis Potosí, Av. Sierra Leona 550, Lomas 2a. Sección, San Luis Potosí 78210, Mexico; 6 Facultad de Ciencias, Universidad Autónoma de San Luis Potosí, Av. Parque Chapultepec 1570, San Luis Potosí 78210, Mexico

**Keywords:** linear epitopes, built-in adjuvant, humoral response, chimeric antigen, COVID-19

## Abstract

Subunit vaccines stand as a leading approach to expanding the current portfolio of vaccines to fight against COVID-19, seeking not only to lower costs but to achieve long-term immunity against variants of concern and have the main attributes that could overcome the limitations of the current vaccines. Herein a chimeric protein targeting S1 and S2 epitopes, called LTp50, was designed as a convenient approach to induce humoral responses against SARS-CoV-2. LTp50 was produced in recombinant *Escherichia coli* using a conventional pET vector, recovering the expected antigen in the insoluble fraction. LTp50 was purified by chromatography (purity > 90%). The solubilization and refolding stages helped to obtain a stable protein amenable for vaccine formulation. LTp50 was adsorbed onto alum, resulting in a stable formulation whose immunogenic properties were assessed in BALB/c mice. Significant humoral responses against the S protein (BA.5 variant) were detected in mice subjected to three subcutaneous doses (10 µg) of the LTp50/alum formulation. This study opens the path for the vaccine formulation optimization using additional adjuvants to advance in the development of a highly effective anti-COVID-19 vaccine directed against the antigenic regions of the S protein, which are less prone to mutations.

## 1. Introduction

The Coronavirus disease 2019 (COVID-19) was first identified in Wuhan, China, on December 2019; it is caused by the severe acute respiratory syndrome coronavirus (SARS-CoV-2). The World Health Organization (WHO) declared a Public Health Emergency of International Concern on 30 January 2020, and on 11 March 2020, it was announced that the COVID-19 outbreak was a pandemic [1]. Recently (on 5 May 2023), the WHO declared the end of COVID-19 as a global health emergency [2]. Since 30 December 2019, the WHO has reported more than 756 million cases, 6.8 million deaths [3], and it has been estimated that at least 200 million people have been diagnosed with long COVID-19 [4]. This pandemic had devastating effects; however, it also led to an unprecedented development of vaccines with novel technologies [5]. Despite not being a pandemic anymore, COVID-19 still exists and continues to pose serious risks to vulnerable individuals.

The fast-track development, production, and approval of the different COVID-19 vaccines have resulted in at least 13,000 million doses [6] administered worldwide until February 2022, which means that 69% of the world population has received at least one dose of a COVID-19 vaccine. Most of the vaccines target the Spike protein (S), which plays a pivotal role in viral entry to the target cells and can be divided into two main domains: S1 (amino acids 12–680) and S2 (amino acids 685–1273). The Receptor Binding Domain, RBD, (amino acids 319–541) is inside the S1 domain. The vaccines that have been authorized or granted an emergency use are CanSino, Covaxin, Johnson & Johnson, Moderna, Oxford/AstraZeneca, Pfizer/BioNTech, Sinopharm, Sinovac, Sputnik Light, Sputnik V, Novavax, KoviVac/Chumakov, IMBCAMS, KCONVAC, Z2001, Abdala, Soberana Plus and 02, Sanofi/GSK, Corbevax, COVIran Barekat, QazVac, and EpiVacCorona [7,8,9]. Despite the large number of available vaccines, only 26% of people from low-income countries have received at least one dose [6]. The low vaccination rate in these countries highlights the unbalanced development of vaccines in all continents and the need for novel affordable vaccines to target low-income regions [10].

Most of the approved and licensed COVID-19 vaccines are based on mRNA, Adenoviruses, and inactivated virus technologies. However, there has been an important effort to develop subunit vaccines; in fact, some of these vaccines have been approved for emergency use in Latin America, the Middle East, and South Asia. There are at least nine subunit vaccines that have been through phase 2 clinical trials: one is expressed in insect cells [11], four in Chinese ovarian hamster cells [12,13,14,15], two in yeast (*P. pastoris*) [16,17], one in FreeStyle 293 cells [18,19], and one is based on synthetic peptides [17].

Sanofi-Pasteur and GlaxoSmithKline (GSK) (VAT00002) [11], Clover Biopharmaceuticals and GSK [12,20] (SCB-2019), Nanogen Pharmaceuticals Biotechnology [13] (Nanocovax), and the National Institute of Allergy and Infectious Diseases [18,19] (MCV-COV1901) have developed vaccine candidates that are based on recombinant SARS-CoV-2 spike (S) protein. SCB-2019 is based on a version of the S protein that is trimeric (as it exists in the virions) and as adjuvant, either AS03 or CpG/alum. This candidate has gone through clinical trials phase 1 (NCT04405908), 2, and 3, and Clover Biopharmaceuticals has reported an efficiency for this formulation equal to 70% against the Delta variant [21,22]. The vaccine candidate VAT00002 uses AS03 as adjuvant and has also gone through phase 1, 2, and 3 clinical trials (NCT04537208 and NCT047268). The results of the Phase 2 and 3 clinical trials have not been reported, but the interim results show a high level of seroconversion and the generation of neutralizing antibodies. The clinical trial of Nanocovax (NCT04922788) showed that this candidate has a 51.5% efficacy against symptomatic infection with the Delta variant. This formulation uses alum as an adjuvant. The vaccine candidate with a prefusion stabilized version of the S protein (MCV-COV1901) contains CpG-1018 and alum, and the interim data of the phase 1 and 2 clinical trials show that this candidate has a good safety profile and elicits a promising immune response [19].

Four vaccines are based on the Receptor Binding Domain (RBD) as the target antigen. The candidate ZF2001 consists of a dimeric tandem repeat of the RBD that is produced in CHO cells and is adjuvanted with alum. There are no published results of the Phase 3 clinical trial (NCT04646590); however, the Phase 1/2 trials showed that 97% of the individuals had neutralizing antibodies after three doses [23]. The Center for Genetic Engineering and Biotechnology (Cuba) developed the vaccine candidate Abdala (CIGB-66) that consists of a recombinant RBD that is produced in *P. pastoris* [24,25]. The formulation contains alum and has gone through Phase 1, 2, and 3 clinical trials, showing 90% effectiveness against severe COVID-19. The candidate BEVOC2 (Corbevax) consists of a recombinant RBD (also produced in *P. pastoris*) that contains either alum or CpG-1018 as adjuvants [16,26]. This candidate has been through clinical trials Phase 1/2 (CTRI/2020/11/029032), 2/3 (CTRI/2021/06/034014 and CTRI/2021/10/037066), and 3 (CTRI/2021/08/036074). The Finlay Vaccine Institute adopted an interesting approach where the RBD is chemically coupled to the tetanus toxoid and uses alum as an adjuvant [15]. Unlike SOBERANA 02, the antigen is produced in CHO-K1 cells. This candidate has been through phase 1, 2, and 3 clinical trials, but the results have not been published. Finally, there is one candidate (EpiVacCorona) where the antigen is a protein comprising synthetic peptides from the most conserved residues of the S protein, which are covalently linked to the SARS-CoV-2 recombinant N protein (EpiVacCorona) [17]. The N protein is used as a carrier, and the vaccine formulation contains alum. The Phase 2 clinical trial showed that all volunteers seroconverted, although the neutralizing antibodies were low [27].

In this context, the use of a diverse set of expression platforms for subunit vaccines is an important goal, especially considering that under a global health emergency, developing countries, and not only developed ones, should have the capabilities for vaccine manufacturing in GMPc-plants, thus ensuring a proper and equal vaccine coverage. Surprisingly, although *Escherichia coli* is a popular host to produce biopharmaceuticals, COVID-19 subunit vaccines produced in this bacterium have not reached clinical trials despite its potential to produce recombinant antigens rapidly and easily, in terms of its ease of genetic manipulation, rapid production, and the requirement of a low-cost infrastructure when compared to that for mammalian and insect cell cultures [28].

Moreover, the rational design of vaccines is currently viable, which could lead to innovative COVID-19 vaccines. Today, specific epitopes from the S protein can be selected based on their potential to be recognized by sera from COVID-19 convalescent patients and their capacity to induce neutralizing antibodies in animal models. The construction of chimeric proteins carrying such epitopes may result in multi-epitope vaccines able to induce an immune response focused on those targets that result in neutralization, avoiding the presence of immunodominant, non-protective epitopes. The use of a protein as a built-in adjuvant is also an important element that may confer key attributes to a multi-epitope vaccine, providing a higher complexity and thus enhancing immunogenicity while having additional epitopes that could improve the memory and the polarization of the induced immune response [29,30]. The heat-labile enterotoxin B subunit of enterotoxigenic *E. coli* (LTB) can be used as built-in adjuvant given its high immunogenicity and adjuvant properties [31,32].

In the present report, a new COVID-19 multi-epitope vaccine candidate produced in *E. coli* is reported, having as innovative attributes, the targeting of highly conserved epitopes from S1 and S2, which are carried by the heat-labile enterotoxin B subunit, resulting in a chimeric protein called LTp50. The methods for the expression (at flask and bioreactor scales), purification, and folding of LTp50 and its use for the formulation of an alum-adjuvanted vaccine were established. The immunogenicity of the LTp50-based vaccines was also assessed in mice.

## 2. Results

### 2.1. Structural Description of LTp50

**Figure 1:**
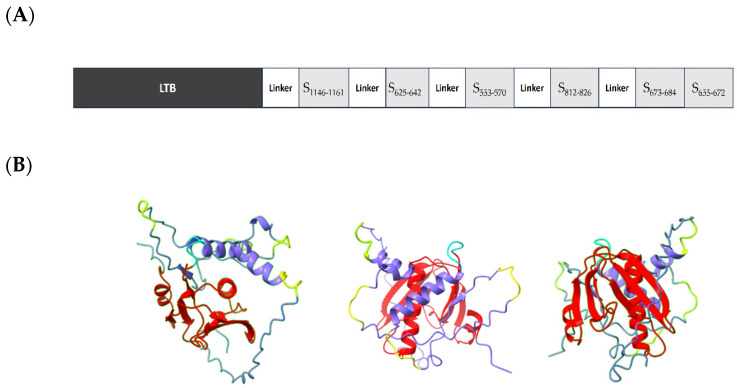
(**A**) Schematic representation of the elements included in the LTp50 chimeric antigen. (**B**) Predicted structure for LTp50. The LTp50 structure predicted shows the classic structure of the heat-labile enterotoxin B subunit of *E. coli*; this structure consists of two sets of three anti-parallel beta sheets and alpha helices (red). The SARS-CoV-2 omicron epitopes (purple) are linked to each other with glycine linkers (yellow) linker. The DSFKEELDKYFKNHTS epitope (S_1146–1161_) forms a four-turn alpha helix, while the DPSKRSFIEDLLFNKV epitope (S_812–826_) is predicted to be a partially ordered alpha helix, the HADQLTPTWRVYSTGSNV epitope (S_625–642_) is mostly unstructured with a small portion that could be forming a beta-sheet. The epitopes TESNKKFLPFQQFGRDIA (S_553–570_), SYQTQTKSHRRA (S_673–684_) and YVNNSYECDIPIGAGICA (S_655–672_) are predicted to be completely unstructured.

The rationale for the design of LTp50 was selecting linear epitopes from S1 and S2 able to induce neutralizing antibodies against the SARS-CoV-2 BA.5 variant, which is currently the most relevant one given its predominancy. Epitope selection was based on two criteria: B-cell epitope prediction using the BepiPred-2.0 server [33] and, in parallel, a literature search to find experimental data supporting notes that the selected epitopes reacted with convalescent COVID-19 sera or induced neutralizing antibodies [34,35,36,37,38]. Although the main goal for the LTp50 is the induction of humoral responses, according to the NetMHCpan-4.0 method some of the selected sequences (epitopes S_553–570_, S_625–642_, S_673–684_, and S_812–826_) potentially bind to MHC I (HLA-A*02:01), which accounts for the immunogenic potential of the construct as these might induce T cell-responses that could provide a Th1/Th2-balanced response. Overall, the chimeric arrangement is intended to potentially induce broad protective, long-lived immune responses against SARS-CoV-2 thanks to the built-in adjuvant and the selected epitopes. LTp50 comprises the full-length sequence of the mature LTB as a built-in adjuvant followed by the selected epitopes, most of them spaced with Gly-Ser linkers (Figure 1A). The structure of the LTp50 chimeric protein was modeled using AlphaFold Colab; while this is a simplified version of AlphaFold v2.3.1, it maintains an accuracy near-identical to the full AlphaFold system [39]. This artificial intelligence software was developed by DeepMind and predicts protein structures to near experimental accuracy by using neural networks. The LTp50 structure predicted shows the classic structure of the heat-labile enterotoxin B subunit of *E. coli*; this structure consists of two sets of three anti-parallel beta sheets and alpha helices (Figure 1B). The SARS-CoV-2 omicron epitopes are linked to each other with either a proline or a glycine (yellow) linker. The DSFKEELDKYFKNHTS epitope (S_1146–1161_) forms a four-turn alpha helix, while the DPSKRSFIEDLLFNKV epitope (S_812–826_) is predicted to be a partially ordered alpha helix, the HADQLTPTWRVYSTGSNV epitope (S_625–642_) is mostly unstructured with a small portion that could be forming a beta-sheet, the epitopes TESNKKFLPFQQFGRDIA (S_553–570_), SYQTQTKSHRRA (S_673–684_), and YVNNSYECDIPIGAGICA are predicted to be completely unstructured. The structure predicted suggests that the SYQTQTKSHRRA (S_673–684_) and YVNNSYECDIPIGAGICA (S_655–672_), epitopes could hide part of the LTB; however, given the fact that this epitope is completely disordered, it is likely that the three largest beta sheets are not really buried by this part of the protein.

### 2.2. Production and Purification of LTp50

**Figure 2:**
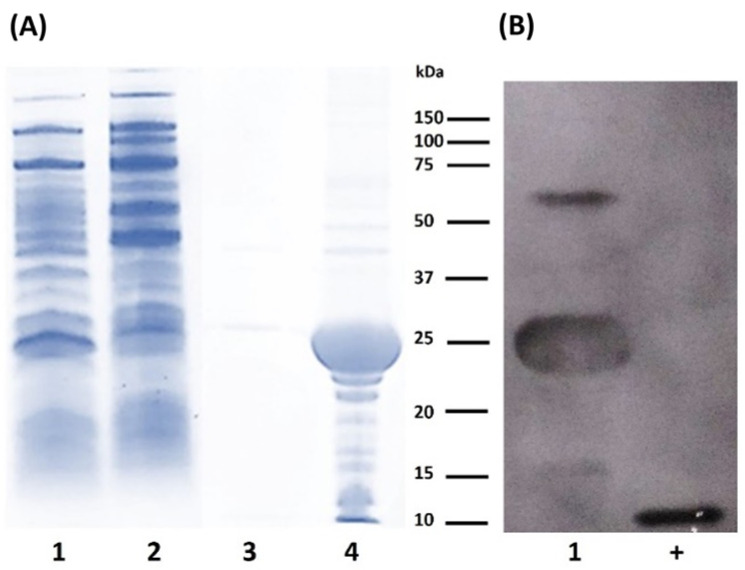
Analysis of protein extracts recovered after flask expression of LTp50. (**A**) SDS-PAGE. Lanes: 1, uninduced soluble fraction; 2, soluble fraction from induced culture; 3, insoluble fraction from uninduced culture; and 4, insoluble fraction from induced culture. (**B**) Western blot. Lanes: 1, insoluble fraction from induced culture; +, positive control (100 ng of recombinant LTB). The theoretical molecular weight for LTp50 sequence is 25.4 kDa.

The expression of LTp50 was conducted at the flask scale to evaluate the conditions that allow efficient production of the chimeric protein, either in the soluble or insoluble fraction. Figure 2 shows the protein patterns for the soluble and insoluble protein extracts obtained during the production at the flask scale. Analysis by either SDS-PAGE (Figure 2A) or Western blot (Figure 2B) demonstrated that the antigenic protein was obtained as inclusion bodies since it is only detected in the insoluble fraction.

**Figure 3:**
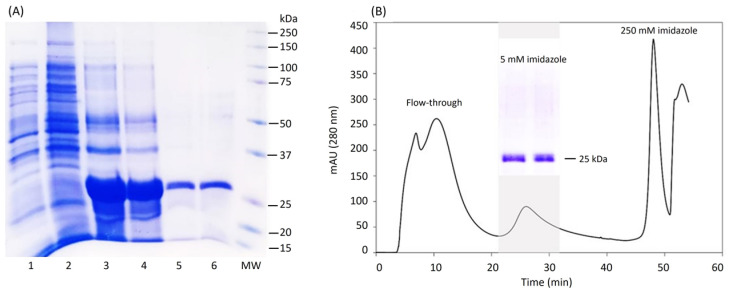
Downstream processing of LTp50. (**A**) Analysis by SDS-PAGE of the recovery phases of the LTp50 protein. Lanes: 1, extract from uninduced culture; 2, soluble fraction from induced culture; 3, supernatant recovered from the penultimate solubilization stage; 4, supernatant recovered from the last solubilization stage and used for chromatography; 5, mixture of the positive fractions recovered after purification of the protein by IMAC; 6, LTp50 after the downstream process; and MW: molecular weight marker. The theoretical molecular weight for LTp50 sequence is 25.4 kDa. (**B**) Chromatogram obtained after injecting S4 to an IMAC column, the LTp50 protein was recovered after increasing the imidazole concentration to 5 mM.

After achieving an efficient expression of LTp50 in the insoluble fraction, the downstream processing was established, starting with the recovery of the protein from the inclusion bodies, followed by different washing and solubilization steps. The solution generated from the last solubilization step (20 mM phosphate buffer, 500 mM NaCl, 8 M urea, and pH 7.4) was used for purification by IMAC. It should be noted that these stages of the protein recovery process were performed under strict conditions of asepsis, hoping to detect low levels of endotoxin in the purified protein. Figure 3 shows the analysis of the protein at the different stages of the downstream process. The endotoxin levels detected in the purified LTp50 protein were 2.31 EU/mL, which meets the permissible limit for this type of contaminant in pharmaceutical products [40].

**Figure 4:**
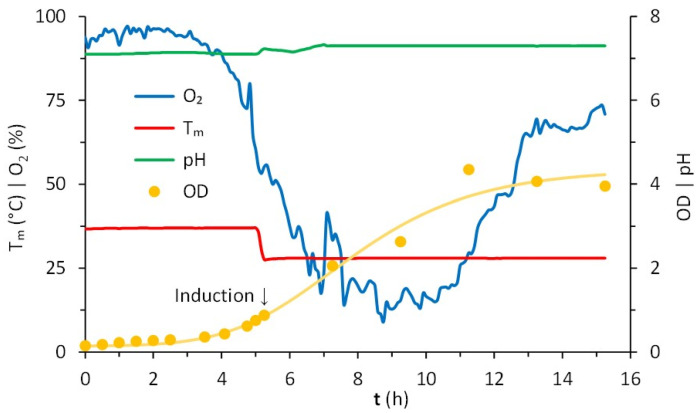
Cultivation in batch of *E. coli* Rosetta in Luria–Bertani (LB) broth. Conditions registered in a bioreactor continuously stirred at 400 rpm, initially with 1.4 L at 37 °C, 95 ± 1.5% of dissolved oxygen, pH of 7.1. Starting with OD = 0.148, the induction occurred after 5.25 h (OD = 0.879) at 28 °C, continuing for 10 h. The consumption of oxygen corresponded to the lag, exponential, and stationary phases of growth.

The production of LTp50 was further scaled-up in a 2-L jar bioreactor. The operational conditions of the upstream processing are represented in Figure 4. Overall, the growth of the *E. coli* Rosetta expression clone in this stirred batch bioreactor using LB broth was favorable. Before the inoculation step, the reactor containing 1.4 L of broth was sterilized, stabilized at 37 °C, and aerated overnight to saturate the medium, reaching a maximum dissolved oxygen concentration of 95 ± 1.5%. Once inoculated, the pH remained controlled at 7.1 and the temperature at 37 °C for 5 h before induction. After the culture reached an OD of 0.755, the temperature was set to 28 °C. Induction started when the culture reached an OD value of 0.879, observing a slight increase in the pH value, which was successfully controlled at 7.3. The analysis of the samples withdrawn at 2 h intervals in the 10 h fermentation period confirms the increase in cell density, followed by a progression to a stationary phase, reaching an average OD value of 4.1 ± 0.2.

**Figure 5:**
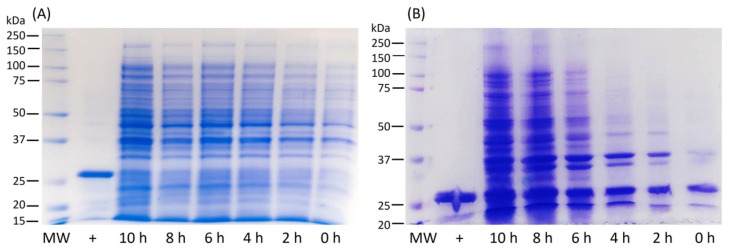
Analysis by SDS-PAGE of total protein extracts obtained at different expression times of the LTp50 protein in a 1.5 L culture using a batch, stirred tank bioreactor. (**A**) soluble fraction, (**B**) insoluble fraction, MW: molecular weight marker, +: LTp50 protein recovered in the flask scale. The theoretical molecular weight for the LTp50 sequence is 25.4 kDa.

SDS-PAGE analysis demonstrated the expression of LTp50 in the insoluble fraction (Figure 5). The oxygen concentration remained stable during the lag phase, decreasing steadily during the exponential phase, wherein the cells grew with a specific growth rate of 1.3 h^−1^. The oxygen concentration increased after such a phase and became stable in the stationary phase.

**Figure 6:**
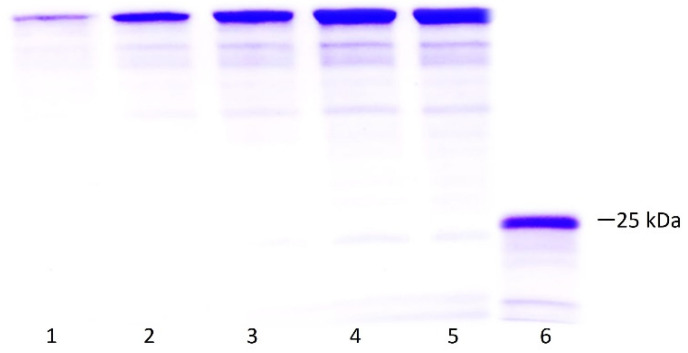
Densitometry to determine purity and concentration of LTp50. Lanes: 1–5, BSA standard curve (1, 2, 3, 4, 5 µg/lane); 6, mixture of the positive fractions recovered after purification of the protein by IMAC. The theoretical molecular weight for the LTp50 sequence is 25.4 kDa.

Figure 6 presents the SDS-PAGE results from which densitometry was performed to calculate the concentration of the protein after the entire recovery process using the ImageJ software. The results of the pure protein yield under the established process are summarized in Table 1. No significant protein was lost during the refolding process.

### 2.3. LTp50 Formulation with Alum

**Figure 7:**
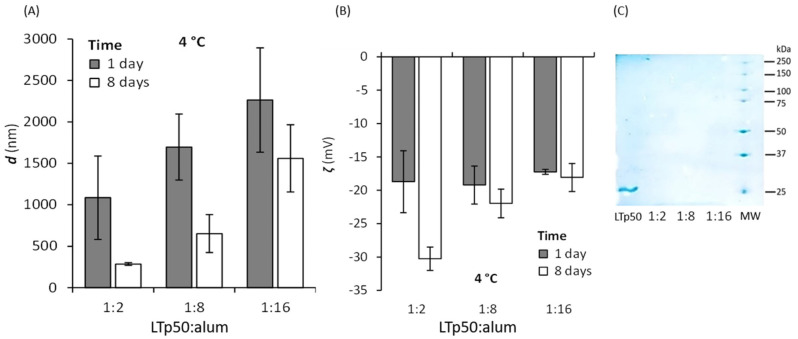
Assessment of the LTp50 adsorption onto alum. The LTp50 antigen was incubated with different LTp50:alum mass ratios (1:2, 1:8, and 1:16) at 4 °C after 1 and 8 days, and a DLS analysis was performed to measure the hydrodynamic diameter (**A**) and ζ potential (**B**) of the antigen-adjuvant complexes. Antigen adsorption was verified by measuring the protein remnants in the supernatant (for all LTp50:alum ratios) after an electrophoretic analysis (**C**), showing almost complete protein adsorption. The first lane shows the soluble LTp50 (which was not contacted with alum), and the last lane is the molecular weight marker.

LTp50 was further applied to formulate a vaccine composed of this target antigen and alum as an adjuvant, using 10% sucrose plus 0.01% Tween-20 as the vehicle. The formulation was validated by monitoring the amount of protein adsorbed onto alum particles using different LTp50:alum ratios, revealing that highly efficient adsorption occurs for all ratios studied (Figure 7C) and the absorbance of the supernatants also corroborated this. Moreover, changes in hydrodynamic diameter and ζ potential were determined after contacting the protein and adjuvant for 1 and 8 days (Figure 7). The LTp50:alum ratios of 1:2 and 1:8 showed a decrease in size after 8 days, while the 1:16 ratio did not. Moreover, the ζ potential did not change from 1 to 8 days for the 1:8 and 1:16 ratios, showing good electrostatic stability (the protein molecules will also impart steric stabilization, contributing to the overall stability of the LTp50-alum complex).

### 2.4. Assessment of LTp50 Immunogenicity

**Figure 8:**
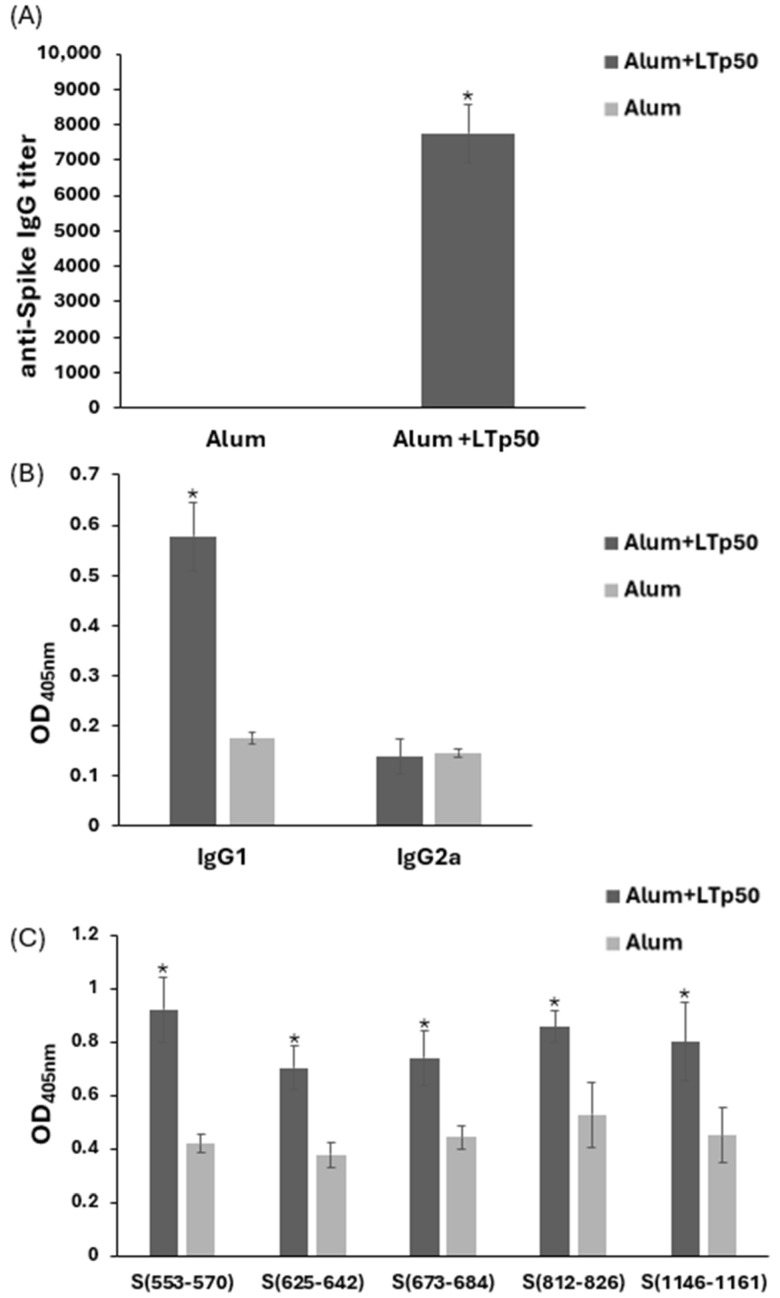
Anti-Spike IgG responses induced in mice by LTp50. Anti-S protein antibody levels were measured in serial dilutions from mice sera immunized with 10 µg doses of LTp50 plus alum or the vehicle plus alum. Mice were subjected to s.c. immunizations on days 1, 14, and 28, and sera were analyzed on day 45. Total IgG levels (**A**) or the IgG1/IgG2a subclasses (**B**) were determined using appropriate secondary antibodies. Reactivity of the test sera was assessed against the individual epitopes from the LTp50 antigen (**C**). The asterisks (* *p* < 0.05) indicate statistically significant differences versus the groups treated with alum alone.

The immunogenicity of the LTp50-alum vaccine was further explored in BALB/c mice. As shown in Figure 8A, the immunization scheme using 10 µg doses of LTp50 led to the development of an anti-Spike serum IgG response with a mean titer value of 7750. An analysis of the IgG subclasses revealed a predominance of the IgG1 subclass (mean value for IgG2a/IgG1 ratio: 0.241), which is associated with a Th2-type (humoral) response (Figure 8B).

**Figure 9:**
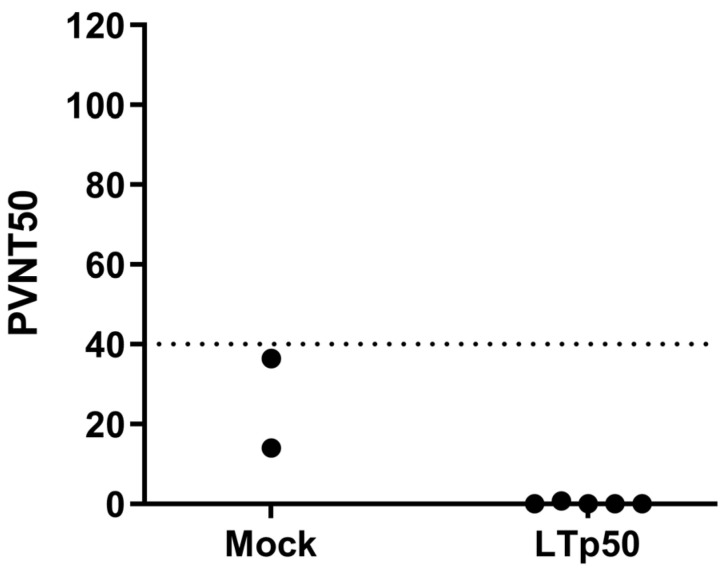
Neutralization assay using SARS-CoV-2 BA.4/5 S-pseudotyped lentivirus. Serial two-fold dilutions of sera were mixed with 1 × 10^6^ relative light units (RLU) of BA.4/5-pseudotyped lentivirus before the addition of HEK293T-hACE2 cells. A virus pre-mixed with media alone (no serum) was used as a positive control to define 0% neutralization while cell-only wells were used as negative controls to define 100% neutralization. After 48 h, Bright-Glo substrate was added to each well and chemiluminescent signals were read using a Synergy HTX microplate reader. PVNT50 values were calculated in the Graphpad Prism 8.0 software using the reciprocal of the dilution that resulted in 50% neutralization. The dotted line indicates the negative cut-off value of 40.

To initially assess the efficacy of the humoral response in neutralizing SARS-CoV-2, sera were tested in a neutralization assay using SARS-CoV-2 BA.4/5 S-pseudotyped lentivirus carrying a luciferase reporter. However, no significant levels of neutralizing antibodies were observed in the mice groups treated with LTp50 (Figure 9), observing readings that are below the cutoff value (PVNT50 < 40) for this assay.

## 3. Discussion

Herein, a new subunit vaccine candidate against SARS-CoV-2, based on the antigen called LTp50, was designed and produced in *E. coli* as an expression host. LTp50 carries S1- and S2-epitopes, under the hypothesis that these regions are less prone to mutations when compared to RBD, which is the target in many vaccine candidates. SARS-CoV-2 has shown the ability to escape from antibodies, especially those that abolish the RBD-ACE2 interaction, through mutations in RBD (which occur in the target binding epitopes or other regions that, upon mutation, affect RBD recognition). Therefore, the efficacy of the vaccines focused on inducing anti-RBD antibodies could be affected by these events. In contrast, the use of epitope-based vaccines focused on non-RBD epitopes that are conserved among variants offers the possibility of generating highly efficacious vaccines in the long-term as the impact of natural mutations could be minimized [41]. Moreover, a substantial number of current COVID-19 vaccines are based on adenovirus vectors, which implies that preexisting immunity against such vectors decreases the efficacy of the vaccine [42]; or RNA encapsulated in liposomes, which often require very low storage temperature [43]. Our LTp50-based formulation lacks this drawback of low efficacy associated to preexisting immunity and offers the advantage that LTB has been already tested in clinical trials for other vaccines, which accounts for the safety of the formulation [44]. Moreover, the stability of subunit vaccines often allows for them to be stored in reasonable temperatures that can be easily ensured under the standard cold chain for vaccine supply.

The LTp50 antigen was efficiently expressed in recombinant *E. coli* and accumulated as inclusion bodies, which implied the standardization of the refolding process to render a soluble, functional protein. The LTp50 yield in terms of pure protein obtained per liter of culture was 22.5 mg/L, which is considered competitive when compared to other reports [45]. Standardizing the purification of the recombinant antigen under a tag-free approach, which is a requirement from a regulatory point of view, is often a challenge. In our case, the implemented chromatographic method allowed for reaching appropriate purity (>90%) and yield to formulate vaccines for human use. The expression and purification methods implemented allowed the production of appropriate amounts of antigen for further characterization of the immune response induced by this candidate in the pertinent animal models.

The main recombinant protein production platforms currently used comprise prokaryotic (e.g., *E. coli* and *Bacillus subtilis*), eukaryotic (e.g., yeast, insect, and mammalian cells), and cell-free systems. *E. coli* is the first-choice host for the initial screening of the target protein production since this organism can be genetically engineered rapidly; it is cultured inexpensively and has a very short duplication time that results in a fast production pipeline. Moreover, several well-characterized strains, vectors, and tags are available for this host to overcome some of the difficulties that can arise for certain proteins (codon bias, inclusion body formation, toxicity, protein inactivity, mRNA instability, and lack of post-translational modifications) [28,46].

At the industrial level, the main advantages of using *E. coli* as an expression host are the use of low-cost culture media, simple scale-up, and low investment in infrastructure required for production under GMPc processes compared to other conventional platforms, such as those produced in mammalian and insect cells [47]. The *E. coli* expression system has been widely examined, but protein expression and purification protocols under this system are labor-intensive and time-consuming. Thus far, of the several reports on *E. coli*-based anti-SARS-CoV-2 vaccines found in the literature, most of them focused on RBD as the target antigen and with the N protein as an alternative [45]. Surprisingly, *E. coli*-made anti-SARS-CoV-2 vaccines have not reached the market yet, highlighting the need for continuing the development of such candidates to reinforce the vaccine production capabilities in developing countries.

The immunogenicity assessment conducted in mice revealed that LTp50 can trigger IgG responses against the full-length S antigen after the administration of three s.c. doses, which accounts for the potential of this candidate to serve as an effective vaccine, providing protective humoral responses. The immunogenicity observed for the LTp50-based vaccine formulation is attributed to the adjuvant activity exerted by the LTB used as a built-in adjuvant and the action of alum as a particulate adjuvant. Since LTB binds specifically to GM1 receptors present in many cell types, it can stimulate immune responses via signal transduction events related to the proliferation of T-cells, an increase in cytokine release, an augment in mucosal/systemic antibody responses, and enhancement of antigen-presenting cells [48]; it also induces B and T cells clustering and delays/arrests T-cell division following endocytosis or B-cell receptor (BCR) uptake of antigen in a ganglioside-mediated manner [49]. LTB has been used as a protein partner (built-in adjuvant) in oral and injectable vaccines. The use of LTB as a co-administered adjuvant has led to similar humoral responses to those induced by alum-adjuvanted formulations with the absence of toxic effects when used at doses up to 50 μg [31]. LTB has also been used as built-in adjuvant in a vaccine prototype based on epitopes of the hepatitis B virus surface antigen, aiding in the induction of high systemic IgG levels [32]. Therefore, fusing LTB to unrelated epitopes is an attractive approach to applying this built-in adjuvant in injectable vaccines, which is a simple and low-cost approach in vaccine development. Alum is a widely used adjuvant in human vaccines formulations, having the ability to efficiently adsorb antigenic proteins to favor a proper pharmacokinetic behavior and enhance immunogenicity [50]. The formulation based on LTp50 adsorbed onto alum particles was validated by confirming that the antigen absorption occurs efficiently and that the conjugates obtained are highly stable after storage at 4 °C for up to 1 week.

Recently, the expression of two chimeric proteins carrying the epitopes TESNKKFLPFQQFGRDIAD (S_553–571_) and DPSKPSKRSFIEDLLFNKVT (S_808–827_) (epitopes that cover some of the epitopes included in LTp50) has been reported [51]. The fusion proteins contain tandem repeats, along with the his-tag for purification. The immunogenic potential was assessed in rabbits but using Freund’s adjuvant for priming, which is not acceptable for human use [51]. Our work, based on tag-free purification and the use of an adjuvant approved for human use, will strengthen our development without considering the inclusion of six different epitopes, which offer the potential to achieve a broad immunity.

Humoral response against the S protein is one of the critical correlates of protection against COVID-19. Protective antibodies exert their action by directly inhibiting viral entry to host cells (neutralization) and post-binding inhibition (effector functions). The former results in the blockade of the early steps of the viral replication cycle by impeding the binding of the S protein to receptors or coreceptors; or by modifying the conformation of the S protein and blocking membrane fusion. Alternatively, post-binding inhibition effector mechanisms achieve the elimination of infected cells by the antibody-mediated effector mechanism, involving antibody-dependent cell cytotoxicity (ADCC), antibody-dependent cellular phagocytosis (ADCP), and antibody-mediated complement-dependent cytotoxicity (CDC). These mechanisms imply the binding of the Fc area to specific Fcγ receptors (FcγR) present on the membrane of natural killer (NK) cells, macrophages, and neutrophils [52]. Antibody-enhanced disease (AED) refers to an immunopathological condition provoked by the presence of pre-existing antibodies against a pathogen that does not neutralize the virus [53]. These antibodies can be generated either during infection or through vaccination. There is evidence that infections with the Feline infectious peritonitis virus, MERS-CoV, and SARS-CoV can result in AED, ADE (antibody-dependent enhancement) and/or vaccine-antibody-enhanced disease (VAED) [54]. Furthermore, some in vitro experiments and infections of animal models with SARS-CoV-2 suggest, based on the Th2 immune response, that infection could result in ADE or VAED. Nonetheless, there is no evidence that antibodies against SARS-CoV-2 can cause VAED in several animal models [55,56]. More importantly, there is no evidence that any of the approved SARS-CoV-2 vaccines could cause VAED in humans [54].

Although no obvious signs of toxicity were observed in the immunization trial performed, such as death and weight lost, there is still a need to assess the safety of the vaccine in an appropriate animal model under GLPs; especially considering that some of the formulations containing CT, which is closely related to LTB, have been associated with hypersensitivity reactions [57]. However, it should be considered that LTB lacks the toxic enzymatic activity since it resides in the A subunit of LT (LTA).

Although our vaccine candidate was unable to induce antibodies capable of exerting neutralization in the pseudovirus-based in vitro assay despite the use of alum as adjuvant and LTB as built-in adjuvant, it should be considered that the antibodies induced by our vaccine could exert other effector mechanisms in vivo distinct to viral entry blockade, such as the elimination of infected cells by ADCC, ADCP, and CDC. Considering that the selected S1- and S2-epitopes are highly conserved among the SARS-CoV-2 VOCs and have been recently described as promising linear epitopes able to induce neutralizing antibodies, targeting non-RBD S1 and S2 epitopes may result in highly effective vaccines that are less susceptible to immune escape [34,35,36,37,38]. Moreover, it has been reported that antibodies recognizing the epitopes included in the LTp50 design have also been detected in convalescent COVID-19 patients [34,35,36,37,38]. Therefore, some avenues are currently under exploration to enhance the efficacy of the LTp50 vaccine in terms of inducing higher titers of antibodies that result in a measurable neutralizing activity using in vitro assays. Our initial focus of using alum alone was based on the principle of using a low-cost, patent-free adjuvant that would guarantee global access to the formulation; however, we are currently exploring different adjuvants (i.e., CpG deoxynucleotides, which strongly activate B cells and elicit TLR9-dependent NF-κB signaling) and evaluating higher antigen doses, seeking to reach higher antibody titers in a two-dose scheme and reevaluating if, under these conditions, the neutralizing activity, in the assay to measure virus entry blockade, can be achieved.

In conclusion, the production system established for the *E. coli*-made LTp50 antigen constitutes a promising tool for obtaining recombinant antigens targeting emerging pathogens in developing countries, which demand an autonomous vaccine production chain using viable platforms in such settings. The optimization of the LTp50 formulation with next-generation adjuvants is guaranteed.

## 4. Materials and Methods

### 4.1. Gene Design and Expression Vector Construction

The LTp50 protein comprises as built-in adjuvant the full-length sequence of the mature heat labile B subunit from enterotoxigenic *E. coli* (LTB, GenBank Acc. No. WP_024168673) fused to six epitopes from S1 and S2 (S_553–570_, S_625–642_, S_655–672_, S_673–684_, S_812–826_, and S_1146–1161_) of the BA.5 variant (GenBank Acc. No. UOZ45804.1). Epitope selection was aided using the BepiPred-2.0 server and the NetMHCpan-4.0 software with the HLA-A*02:01 allele. GGGGS linkers were placed between the target epitopes, whereas a proline-containing linker was placed between LTB and the S epitopes. No tags were included in the construct to avoid regulatory issues associated with unrelated sequences in the biopharmaceutical that might trigger undesired immune responses. The synthetic gene coding LTp50 was obtained by Biomatik Co. (ONT, Canada) following a codon optimization process, and it was cloned at the NcoI and BamHI sites in the pET15b vector. The expression vector was verified by restriction patterns and conventional sequencing (Sanger-based method). The pET15b-LTp50 vector was purified from TOP10 *E. coli* cultures, and it was transferred to the *E. coli* Rosetta strain using chemically competent cells by conventional methods. Luria Bertani plates supplemented with ampicillin (100 mg/L) and chloramphenicol (40 mg/L) were used for selection purposes.

### 4.2. Protein Expression in Flasks

The expression of LTp50 was initially explored using small-scale flasks. For this purpose, a seed culture was prepared with a clone carrying the pET15b-LTp50 construct by inoculating it into 50 mL of LB medium (10 g/L bacto-peptone, 5 g/L yeast extract, and 10 g/L NaCl) supplemented with ampicillin (100 mg/L) and chloramphenicol (40 mg/L), followed by overnight incubation at 37 °C under orbital shaking. A total volume of 1 L of LB medium supplemented with antibiotics was divided into four 1 L flasks, which were inoculated with the seed culture (10% *v*/*v*) and incubated at 37 °C under orbital shaking until an OD_600_ = 0.8 was reached. The culture temperature was set to 28 °C, and lactose was subsequently added at 1.5 g/L to induce the expression of the LTp50 protein. The expression phase was performed at 28 °C for 16 h. The endpoint cultures were centrifuged at 7000× *g* rpm for 10 min at 4 °C, and the pellets were stored at −40 °C for further analysis.

### 4.3. Protein Expression in Bioreactor

The scale-up of the LTp50 production process was explored in a batch-stirred tank bioreactor. Seed culture was prepared in four 1 L flasks containing 200 mL of LB medium plus antibiotics; the medium was inoculated with the expression clone, and incubation happened overnight at 37 °C under orbital shaking. The cells were harvested by centrifugation and resuspended in 20 mL of fresh LB medium. Batch cultures were grown in a 4 L jar fermenter (ez-Control system model 56,156, Applikon Biotechnology, Delft, The Netherlands) containing 3.5 L of LB medium with ampicillin (100 mg/L) and chloramphenicol (40 mg/L). Upon inoculation, an OD_600_ = 0.6 was reached, and the following expression conditions were set up: pH at 7.0 ± 0.5 by adding 2 M HCl or 2 M NaOH, and O_2_ saturation above 40% by culture stirring (400–600 rpm) and aeration (0.5–1 L/min). The temperature was set to 37 °C until the optical density was close to 0.8 and, subsequently, set to 28 °C. Once the expression temperature was reached, lactose was added at 1.5 g/L to begin the expression phase, which was performed during 10 h. 10 mL culture samples were collected at 2 h intervals. All samples recovered were centrifuged at 7000× *g* rpm for 10 min at 4 °C, and pellets were stored at −40 °C.

### 4.4. Protein Extraction and Purification

Bacterial biomass recovered from either culture fermentation (flask or bioreactor) was subjected to a cell disruption protocol through osmotic shock followed by sonication. In brief, the harvested cells were resuspended in a cold hypertonic solution (100 mM Tris-HCl, 20% (*w*/*v*) sucrose, and pH 7.4) using 10 mL per gram of bacterial biomass. The extract was centrifuged at 7000× *g* rpm for 10 min at 4 °C, and the pellet was washed and resuspended in a hypotonic solution (injectable water plus 0.01 mM PMSF) using twice the volume employed in the previous step. The suspension was kept on ice and subjected to 6–9 ultrasonication cycles (30 s on/30 s off) using a GEX130PB device (Twinsburg, OH, USA) at a 70% amplitude. The extract was centrifuged at 7000× *g* rpm for 10 min at 4 °C to recover the insoluble fraction (inclusion bodies) and soluble proteins (soluble fraction). These fractions were stored at −40 °C until further analysis. The inclusion bodies containing LTp50 were subjected to a washing procedure followed by a solubilization protocol using the buffers and conditions summarized in Table 2. The buffers used in each of the steps were added at 5% (*v*/*v*) (with respect to the total culture volume), maintaining the suspension/solutions on ice during the incubation periods. Each step was followed by centrifugation at 7000× *g* rpm for 10 min at 4 °C, and the supernatant obtained from the last solubilization step was recovered and stored at −40 °C.

LTp50 from inclusion bodies was purified using immobilized metal affinity chromatography (IMAC). For this, the insoluble fraction previously washed was solubilized using buffer A (20 mM phosphate, 500 mM NaCl, 8 M urea, pH 7.4). 1 mL of this sample (after removing insoluble material at 13,300× *g* for 2 min) was injected onto an IMAC column (2 mL), previously equilibrated with buffer A and loaded with Ni^2+^ ions, at a flow rate of 0.25 mL/min. The recombinant protein was recovered after increasing the imidazole concentration to 5 mM. Highly pure fractions were collected and subjected to protein refolding using dialysis with three sequential buffers: PBS + 4 M urea at pH 7.0, 50 mM sodium carbonate + 0.01% Tween 20 + 10% glycerol at pH 9.2, and 10% sucrose + 0.01% Tween 20. Purified and refolded LTp50 was concentrated using a spin column. The concentrated sample was kept at −20 °C until further use. SDS-PAGE was used to quantify purity and protein concentration using densitometry with Image J 1.47a [58].

### 4.5. Protein Analysis Protocols

Protein samples were analyzed by SDS-PAGE using a 5× reducing dye buffer with denaturing conditions (10 min under boiling water). 10% polyacrylamide gels were used and subjected to conventional Coomassie blue staining. Immunodetection of LTp50 was performed by Western blot following a protocol previously described. Briefly, the gels were blotted onto a nitrocellulose membrane, which was subsequently blocked with fat-free milk and labeled with a mouse anti-serum recognizing the LTB moiety (1:1000 dilution). Recombinant LTB was used as positive control (Sigma-Aldrich, St. Louis, MO, USA). Detection was carried out using a goat horseradish peroxidase-conjugated secondary anti-IgG mouse antibody in combination with the SuperSignal West Pico chemiluminescent substrate (Pierce, Rockford, IL, USA) [59]. Endotoxin quantification was performed using the ToxinSensor Chromogenic LAL Endotoxin Assay Kit following the supplier’s instructions (GenScript, Piscataway, NJ, USA).

### 4.6. Vaccine Formulation

The vaccine was formulated using LTp50:alum mass ratios of 1:2, 1:8, and 1:16. The antigen mass was fixed to 10 µg, and the respective alum mass was obtained from a suspension having a concentration of 10 mg/mL. The mixture was contacted for 1 h at 4 °C, followed by immunization. The vehicle was 10% sucrose plus 0.01% Tween-20. To corroborate that the antigen was successfully adsorbed by the alum particles, the supernatant of the vaccine was analyzed by SDS-PAGE. Moreover, this supernatant was also analyzed by measuring absorbance at 280 nm. Finally, changes in the hydrodynamic diameter and ζ potential of the vaccine formulations were determined after keeping the vaccine for 1 and 8 days at 4 °C using a dynamic light scattering device (Malvern).

### 4.7. Immunogenicity Assessment

The immunogenicity assessment for LTp50 was approved by the Institutional Ethics Committee (CEID-2020-07R1). Experimental BALB/c mice groups were established (*n* = 5, 12-week-old) to perform a dose-response experiment. Mice were immunized by the subcutaneous (s.c.) route on days 1, 14, and 28 with one of the following formulations: 0.1 µg of LTp50 plus alum (80 µg, G biosciences, cat no. 786-1215), 2.5 µg of LTp50 plus alum (80 µg), and 10 µg of LTp50 plus alum (80 µg). Once the dose leading to the higher anti-S antibody response was identified, a second immunization scheme under the same conditions was performed but with larger test groups (*n* = 10, 5 males and 5 females). One group was immunized with alum alone (80 µg) as negative control, and the second group was immunized with 10 µg of LTp50 plus alum (80 µg).

Blood samples were obtained on day 45 to obtain sera, which were analyzed in triplicate by ELISA to titrate anti-S protein binding antibodies using 1/2 serial dilutions. Sera preparation and ELISA were performed according to previously reported methods [60]. Briefly, 96-well polystyrene plates were coated with the S protein from the BA.4/BA.5/BA.5.2 variant (Sino Biological Inc. cat. no. 40589-V08H32, 80 ng/well) diluted in a carbonate buffer (15 mM Na_2_CO_3_ and 35 mM NaHCO_3_) and incubated overnight at 4 °C. The plates were washed three times with PBS-T followed by a blocking step with 5% fat-free milk at 25 °C for 2 h. After washing, serial dilutions of the test sera were added, followed by an overnight incubation at 4 °C. Afterward, horseradish peroxidase-conjugated secondary antibodies (anti-mouse IgG or anti-mouse IgG1/IgG2a subclasses) were added at a 1:10,000 dilution in PBS. After a final washing step, an ABTS substrate solution containing 0.6 mM 2,2′-azino-bis (3-ethylbenzothiazoline-6-sulfonic acid) (ABTS; Sigma-Aldrich), 0.1 M citric acid, and 1 mM H_2_O_2_ at pH 4.35 was added. After incubation for 60 min at 25 °C, OD values at 405 nm were recorded with a Multiskan FC microplate photometer (Thermo Fisher Scientific, Waltham, MA, USA). The statistical differences in humoral responses were determined by U Mann–Whitney or Kruskal–Wallis tests using Dunn’s post hoc test. A *p* < 0.05 was considered significant. All data were analyzed and graphed using the GraphPad Prism 8.0 software.

### 4.8. Pseudotyped Virus-Based Neutralization Assay

Firefly luciferase-expressing reporter lentivirus pseudotyped with the SARS-CoV-2 BA.4/5 S were generated in HEK293T cells. Serial two-fold dilutions of sera were analyzed in triplicate by mixing the test sera with 1 × 10^6^ relative light units (RLU) of pseudotyped virus in a total volume of 100 µL and incubated at 37 °C in a humidified 5% CO_2_ incubator for 1 h before 1 × 10^4^ HEK293T-hACE2 cells were added to each well. A virus sample pre-mixed with media alone (no serum) was used as a positive control to define 0% neutralization while cell-only wells were used as negative controls to define 100% neutralization. After 48 h at 37 °C, the Bright-Glo substrate (Promega, Madison, WI, USA) was added to each well and chemiluminescent signals were read using a Synergy HTX microplate reader. PVNT50 values were calculated in a Graphpad Prism 8.0 using the reciprocal of the dilution that resulted in 50% neutralization.

## Figures and Tables

**Table 1:** LTp50 expression data. * (% pure protein LTp50).

Scales	LTp50 in Crude Extract (mg/L Culture)	Pure LT-p50 (mg/L Culture)	Yield *	Purity
Flask (1 L)	33.7 mg/L	16.7 mg/L	25.6%	>90%
Bioreactor (1.5 L)	52.6 mg/L	22.5 mg/L	42.8%	>90%

**Table 2:** Buffers and conditions established for the isolation and solubilization of LTp50 inclusion bodies.

Stage	Buffer	Rounds	Incubation time
Washing	PBS 1×, 1% (*v*/*v*) Triton X-100	2	20 min
Washing	PBS 1×	2	20 min
Washing	20 mM phosphate buffer, 500 mM NaCl, 4 M urea, pH 7.4	1	20 min
Solubilization	20 mM phosphate buffer, 500 mM NaCl, 8 M urea, pH 7.4	1	20 min
Solubilization	20 mM phosphate buffer, 500 mM NaCl, 8 M urea, pH 7.4	1	overnight

## Data Availability

The data presented in this study are available on request from the corresponding author.
